# Anti-Inflammatory Activity of Natural Products

**DOI:** 10.3390/molecules21101321

**Published:** 2016-10-01

**Authors:** Abdullatif Azab, Ahmad Nassar, Abed N. Azab

**Affiliations:** 1 Institute of Applied Research, The Galilee Society, P.O. Box 437, 20200 Shefa-Amr, Israel; abedazab@gal-soc.org; 2 Department of Clinical Biochemistry and Pharmacology, Faculty of Health Sciences, Ben-Gurion University of the Negev, P.O. Box 653, 84105 Beer-Sheva, Israel; nassarah@post.bgu.ac.il; 3 Department of Nursing, Faculty of Health Sciences, Ben-Gurion University of the Negev, P.O. Box 653, 84105 Beer-Sheva, Israel

**Keywords:** natural products, anti-inflammatory activity, plant extract, pure compounds

## Abstract

This article presents highlights of the published literature regarding the anti-inflammatory activities of natural products. Many review articles were published in this regard, however, most of them have presented this important issue from a regional, limited perspective. This paper summarizes the vast range of review and research articles that have reported on the anti-inflammatory effects of extracts and/or pure compounds derived from natural products. Moreover, this review pinpoints some interesting traditionally used medicinal plants that were not investigated yet.

## 1. Introduction

Inflammation usually occurs when infectious microorganisms such as bacteria, viruses or fungi invade the body, reside in particular tissues and/or circulate in the blood [1,2,3]. Inflammation may also happen in response to processes such as tissue injury, cell death, cancer, ischemia and degeneration [1,4,5,6,7,8,9]. Mostly, both the innate immune response as well as the adaptive immune response are involved in the formation of inflammation [1,5,9]. The innate immune system is the foremost defense mechanism against invading microorganisms and cancer cells, involving the activity of various cells including macrophages, mast cells and dendritic cells. The adaptive immune systems involves the activity of more specialized cells such as B and T cells who are responsible for eradicating invading pathogens and cancer cells by producing specific receptors and antibodies. 

Numerous inflammatory mediators are synthetized and secreted during inflammatory responses of different types. Inflammatory substances are usually divided to two main categories: pro- and anti-inflammatory mediators. Nevertheless, some mediators such as interleukin (IL)-12 possess both pro- and anti-inflammatory properties [10]. Among the inflammatory mediators and cellular pathways that have been extensively studied in association with human pathological conditions are cytokines (e.g., interferons, interleukins and tumor necrosis factor α), chemokines (e.g., monocyte chemoattractant protein 1), eicosanoids (e.g., prostaglandins and leukotrienes) and the potent inflammation-modulating transcription factor nuclear factor κ B.

Tumor necrosis factor (TNF)-α is an important pro-inflammatory cytokine which is secreted from various cells and exerts many cellular effects [11,12]. TNF-α has been associated with multiple illness states in humans, including immune and inflammatory diseases, cancer, psychiatric disorders, among others. Another cytokine which mostly exerts a pro-inflammatory activity is IL-1α [13,14]. It stimulates the secretion of pro-inflammatory cytokines such as IL-1β and TNF-α [13,14]. However, IL-1α has also been associated with anti-inflammatory activity. Similar to IL-1α, IL-6 usually acts as a pro-inflammatory cytokine but it also has some anti-inflammatory effects. As mentioned above, the IL-12 family of cytokines (including IL-12, IL-23, IL-27 and IL-35) possess both pro- and anti-inflammatory functions [10,15,16]. On the other hand, IL-10 is a potent anti-inflammatory cytokine the activity of which impedes the action of many pro-inflammatory mediators [17,18,19]. By weakening and controlling the inflammatory response IL-10 helps to maintain tissue homeostasis and attenuates the damage that may result from an exaggerated inflammatory response [17,18,19].

Prostaglandin (PG) E_2_ is probably the most studied PG in association with human physiological and pathological conditions [20]. It has various physiological roles including regulation of normal body temperature, gastric mucosal integrity, renal blood flow and the function of female reproductive system. On the other hand, alterations in PGE_2_ activity are associated with pathological conditions such as inflammatory diseases, abnormal changes in body temperature, colorectal cancer, among others. The pathway of PGs synthesis starts with generation of arachidonic acid from cell membrane phospholipids by phospholipase A2 (PLA2). Then, arachidonic acid is converted to PGs by the enzyme cycloogygenase (COX) [20]. Among the three known COX isoforms (COX-1, COX-2 and COX-3), the inducible enzyme COX-2 is recognized as the most active during inflammatory processes. Leukotrienes (LTs) such as LTB_4_ were also linked to human illness states including inflammation, asthma and depression [21,22,23]. LTs are produced by the enzyme 5-lipooxygenase (5-LOX) [22]. Another enzyme that is highly associated with inflammatory conditions is nitric oxide synthase (NOS) which produces nitric oxide (NO) [24]. Similar to COX-2, inducible NOS (iNOS) is the most pro-inflammatory NOS isoform.

The transcription factor nuclear factor κ B (NFκB) is a prominent regulator of immune and inflammatory responses and is highly involved in the pathophysiology of cancer [25,26,27]. In mammals, the NFκB machinery comprises several members (e.g., p50 and p65) which regulate both physiological and pathological processes [25,26]. At resting (un-stimulated) conditions NFκB resides in the cytoplasm [26]. Following activation by various infectious/inflammatory/mitogenic stimuli, NFκB proteins translocate to the nucleus and induce transcription of inflammatory-associated genes [26,27].

The practice of using plants, their parts or extracts as anti-inflammatory compounds is known since antiquity. For example, concentrated, viscous aqueous extract of ripe carob (*Ceratonia siliqua* L.) has been used for decades in Arab folk medicine, especially for treating mouth inflammations [28]. The use of plants or plant products for medicinal purposes was mostly documented in books and, lately, in an enormous number of websites (where the reliability of some of these websites must be examined carefully). In the last decades, hundreds of research and review articles were published regarding the anti-inflammatory activities of plants. In this review we introduce some highlights of the literature published mainly during the last three decades, with a few references to earlier reports. 

## 2. Review Articles of Natural Non-Plant Materials

As mentioned above, dozens of review articles have been published in the last few decades. Interestingly, a notable number of them were published by scholars from India, a country with a well rooted traditional plant medicine and a vast diversity of medicinal plants. Our summary here focuses on some of these reviews, but also includes articles from other parts of the world in order to provide a wider view. This part includes review articles which summarize the anti-inflammatory activities of non-plant natural products which exist in mushrooms and honey. Mushrooms and honey traditional therapies are very well established in most cultures. Moreover, mushrooms/honey mixtures with other plant materials (including various extracts) were used in folk medicines since ancient times.

**Figure 1:**
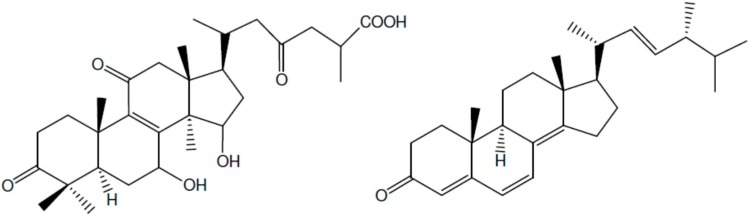
Structures of ganoderic acid A (**A**) and ergosta-4-6-8(14),22-tetraen-3-one (**B**).

One of the early articles that introduced the anti-inflammatory activities of mushrooms and some of their compounds was published by Lindequist et al. in 2005 [29]. Four different mushroom species were reviewed: *Phellinus linteus* that is used in traditional medicines of cultures of East Asia, *Ganoderma lucidum* (Lingzhi mushroom) which also has a long history of medicinal use in China, the widespread *Pleurotus pulmonarius* (subtropical forests) and the edible *Grifola frondosa*. Some biologically active compounds were extracted from each of these mushrooms. For example, eight different triterpenoid ganoderic acids were isolated from *G. lucidum*, but only four of them exerted anti-inflammatory activity (Figure 1A shows one of these compounds). From *G. frondosa*, an ergosterol oxidation product active as an anti-inflammatory agent was isolated (Figure 1B).

**Figure 2:**
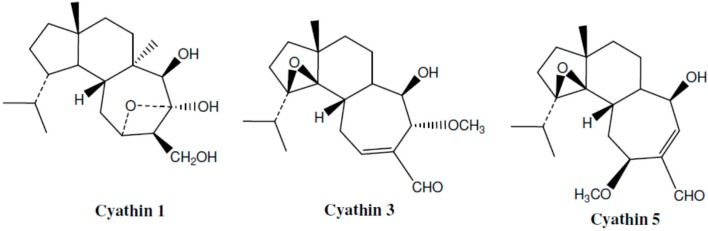
Structure of some cyathins isolated from mushrooms.

An excellent, comprehensive review of anti-inflammatory activities of mushrooms was published by Elsayed and his colleagues in 2014 [30]. This article provides detailed, systematic information about a large number of mushroom species, many biologically active compounds, and importantly, suggested mechanisms of action. Among the most established anti-inflammatory effects of mushrooms that were reported in this article were: reduction of IL-1β, IL-6, LTs, PGs and TNF-α levels, and, inhibition of COX-2, iNOS and NFκB activity [30]. The authors state that terpenoids are the largest group of anti-inflammatory compounds in mushrooms and presented some seven-membered, structurally interesting examples of these compounds (such as cyathins and related compounds, Figure 2).

**Figure 3:**
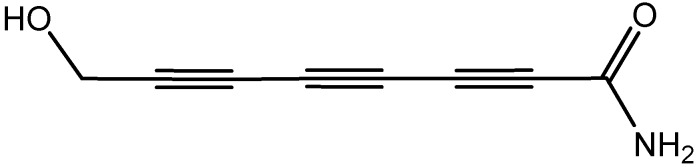
Structure of agrocybin.

In their article, Elsayed et al. [30] addressed a study by Ngai et al. [31] which reported on the isolation of a 15 amino acids peptide from *Agrocybe cylindrace* which the authors named “agrocybin”. Ngai et al. [31] reported that “agrocybin” exerted antifungal but not anti-inflammatory activity. However, for the sake of accuracy, it is important to mention that the name agrocybin also refers to a different compound (not a peptide), reported by Rosa and his colleagues [32] who isolated it from another *Agrocybe* species, *A. perfecta*. To the best of our knowledge, this compound also named agrocybin is a polyeyne amide [32,33], as shown in Figure 3.

The second source of non-plant, natural material with anti-inflammatory activity is honey. Since it is one of the most ancient nutritious foods and was mentioned in most holy religious texts, honey has been used for medicinal purposes since antiquity. Numerous review articles were published about the anti-inflammatory properties of honey. Almost all of these reviews focus on clinical evidence for the anti-inflammatory activity of honey but lack any reporting of active compounds. Moreover, most of the articles indicate that the precise mechanism underlying the anti-inflammatory activity of honey is unknown, although some present proposed mechanisms [34,35]. Mostly, honey was reported to have anti-inflammatory effects (such as reduction of TNF-α levels, attenuation of COX-2 activity, and inhibition of NFκB translocation to the nucleus) but pro-inflammatory actions were also indicated (e.g., elevation of NO production) [34,35].

## 3. Review Articles on Natural Plant Materials

Among the different biological activities of natural plant products that have been published until now, anti-inflammation is one of the most reported effects. Table 1 summarizes selected review articles which report on the anti-inflammatory properties of natural plant materials.

## 4. Active Anti-Inflammatory Plant Extracts, Essential Oils, Juices and Powders

Extracting plant materials is the first major step towards testing the biological activities of this plant. In doing so, there are many advantages and some disadvantages, comparing with isolation of pure active compounds. When a whole extract is used, there is a good chance for synergism between active components that might be lost when each of these components is isolated. Such synergism was discovered in several medicinal tests, including those for anti-inflammatory activity [36,37]. On the contrary, the mixture of different compounds together may also lead to inhibitory effects, namely, that one component may reduce the biological activity of the other. In line with this assumption, some studies have showed that the anti-inflammatory activity of pure compounds (such as amentoflavone, pseudohypericin, and hyperforin, isolated from extracts of *Hypericum perforatum*) is higher than that of the extracts [38]. In addition to plant extracts, essential oils [39,40], plant juices [41] and plant powders [42] are also widely used for medicinal purposes.

Solvent selection for extraction of plant materials is one of the most important factors in determining the potential activity of the extract, since the solvent polarity determines which compounds will be extracted and which will not. For example, it is unlikely that water (very polar) will extract the active anti-inflammatory compound monoterpene 1,8-cineole (*Achillea millefolium*) but will easily extract protocatechuic acid (*Boswellia dalzielii*), and vice versa for *n*-hexane (non-polar). Thus, in many cases of newly studied plants, various extracts are prepared with solvents that have a wide polarity range. Table 2 summarizes selected research articles which have reported on the anti-inflammatory activity of plant extracts. 

There are several worth mentioning points regarding the information presented in Table 2. The plant *Corchorus olitorius*, known as Mulukhiyah in the Middle East, is one of the most important edible plants in this region. Despite this fact there are relatively very few reported studies regarding the medicinal properties of this plant. A study by Zakaria et al. [43] found that it exerted potent anti-inflammatory and antipyretic effects (Table 2). The title of the article by Islam et al. [44] states that “ethanol” was used to prepare extracts from mango (*Mangifera indica*) leaves, however, in the “Materials and Methods” section only methanol was mentioned as the extracting solvent. In the study by Li et al. [45] different extracts were prepared from hawthorn fruit (*Crataegus pinnatifida* Bunge var. *typica* Schneider). A first extract was prepared using 70% methanol in water. Then, this extract was concentrated and extracted again with each of the following solvents: water, ethyl acetate, *n*-butanol and dichloromethane. Only the aqueous extract showed a significant anti-inflammatory activity. Of note, the most abundant hawthorn species in eastern Mediterranean region—*Crataegus aronia*—was never reported, although many of its medicinal activities are well acknowledged. A study by Abu-Gharbieh et al. [46] examined the anti-inflammatory effect of the aqueous extract of *Micromeria fruticosa* in mice. They reported a prominent reduction in carrageenan-induced paw edema. Moreover, pretreatment with the extract led to a significant decrease in gastric mucosal lesions induced by high-dose indomethacin, attesting for a gastro-protective effect of the extract.

Interestingly, *M. fruticosa* is one of the most useful herbs in western Asia, especially in the Middle East. Nevertheless, the specific compound(s) that is/are responsible for its anti-inflammatory activity is/are still unknown. Furthermore, *M. sylvestris* L*.* is an extensively eaten and widely used plant for medicinal purposes in the east Mediterranean region. A similar *Micromeria* species is *M. nicaeenis.* The chemical composition of this plant is unknown and, to the best of our knowledge, its anti-inflammatory activity has not been studied yet. 

**Figure 4:**
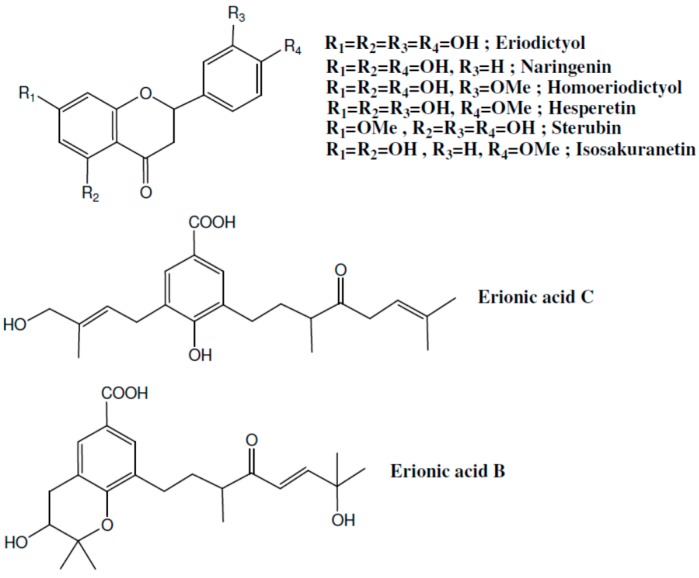
Structure of active anti-inflammatory compounds isolated from *Eriodictyon angustifolium*.

A study by Walker et al. [101] examined the anti-inflammatory properties of *Eriodictyon angustifolium* (a North American shrub) and its major active compounds on LPS-induced inflammation in human gingival fibroblasts. The dried leaves of the plant were extracted and the crude extracts were analyzed. Eight active compounds were identified as shown in Figure 4. Some of the extracts showed a profound anti-inflammatory activity. As mentioned above, aqueous extract of ripe carob (*Ceratonia siliqua*) is among the most used remedies in Arab traditional medicine [28]. A recent study by Lachkar et al. [103] clearly demonstrated that carob exerts prominent anti-inflammatory properties which are comparable to those of the potent anti-inflammatory drug indomethacin. Ripe pods of carob provide food for humans and animals. Ripe pods are traditionally extracted with boiling water after being crushed. The filtered extract is evaporated to viscous, sweet paste. In addition to its nutritional value, this paste has traditional, proven anti-inflammatory qualities, especially regarding mouth inflammations. Thus, it is strange that these qualities are just being studied in the last few years [103,109,110].

## 5. Selected Reports of Single Natural Products with Anti-Inflammatory Activities

As indicated in the previous section, isolation and testing of a single natural product for biological activities has both advantages and disadvantages. Two major advantages that were not mentioned are: (i) Testing a single active compound enables a thorough elucidation and better understanding of its mechanism of action; and (ii) if a single compound proves efficacious, it is possible to perform slight modifications on its structure or produce synthetic analogues in order to obtain more potent/efficacious compounds. In this regard, half of the the 2015 Nobel Prize in medicine was awarded to Campbell and Omura mainly for the synthesis and discovery of the anti-malarial compound ivermectin, which is the result of a very slight modification (a dihydro derivative) of the natural product avermectin [111].

Table 3 summarizes selected reports of anti-inflammatory activity of pure compounds that have been thoroughly investigated so far. An early study by Gupta et al. [112] reported that ursolic acid and cucurbitacin B did not exhibit anti-inflammatory properties. However, the findings concerning ursolic acid [112] are contradicted by later reports [50,75].

**Figure 5:**
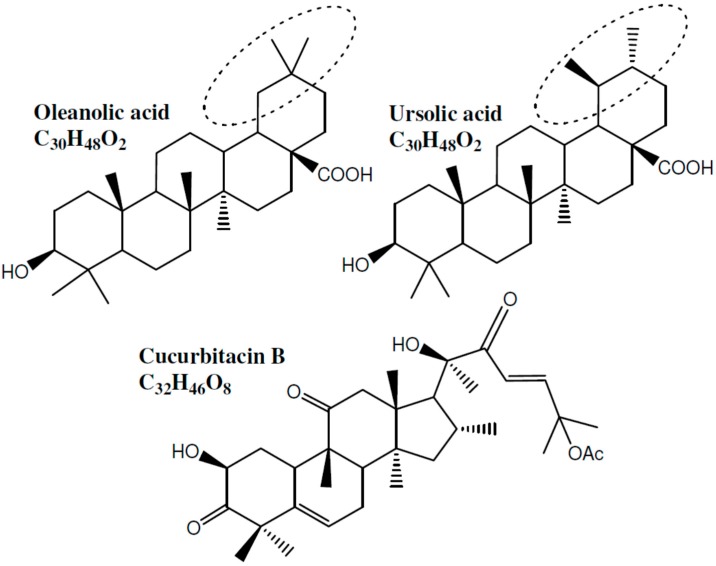
Structures of oleanolic acid, ursolic acid and cucurbitacin B.

Many studies have presented ursolic acid as one of the major compounds responsible for the anti-inflammatory activities of various plants [119,120]. Moreover, as seen in Figure 5, oleanolic acid (which possesses anti-inflammatory effects, Table 3) and ursolic acid are structural isomers with very small difference in their structures. As for cucurbitacin B, similarly, the findings of Gupta et al. [112] contradict later reports which clearly indicated that the anti-inflammatory activity of *Ecballium elaterium* (squirting cucumber) [121,122] and *Cucurbita andreana* [123] is mainly due to this compound.

**Figure 6:**
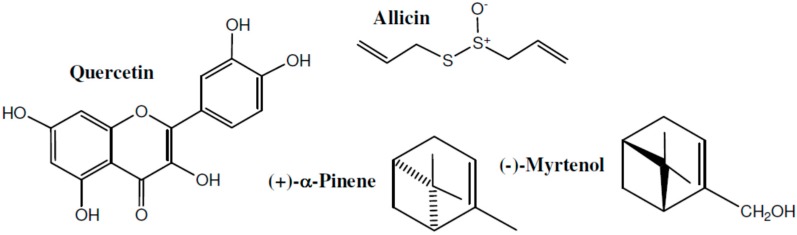
Structures of quercetin, allicin, (+)-α-pinene and (−)-myrtenol.

In a study by Guardia et al. [113] three plant flavonoids—rutin, quercetin and hesperidin—were found to have anti-inflammatory effects. Quercetin is an abundant polyphenol in the plant kingdom. Its structure (with other compounds) is shown in Figure 6. Onions (*Allium cepa*) contain a high concentration of quercetin and studies confirmed the anti-inflammatory activities of onion juice and extracts [124]. *Abutilon indicum* also contains high amounts of quercetin and has significant anti-inflammatory activity [94]. Furthermore, garlic contains large amounts of allicin (the structure of which is shown in Figure 6) which exerts potent anti-inflammatory effects [114]. 

As for the vast majority of natural products, even short term heating of garlic reduces the anti-inflammatory activity of allicin [125]. Another potent anti-inflammatory compound is (−)-myrtenol ([115], Table 3). As seen in Figure 6, it is essentially a mono-oxidized isomer of (−)-α-pinene. Interestingly, the anti-inflammatory activity of (−)-α-pinene is negligible compared with (+)-α-pinene [126], while the anti-inflammatory activity of (+)-myrtenol was never reported. This “enantiomeric selectivity” does not always occur as reported for equal anti-inflammatory activities of the enantiomers shikonin and alkannin found in *Alkanna tinctoria* [127]. A study by Thao et al. [116] examined the anti-inflammatory properties of different terpenes and polyphenols. Twenty six compounds, some of which were novel, were isolated and tested in this research. The most active anti-inflammatory compound was a derivative of juglone (5-hydroxy-7-methyl-2-methoxy-1,4-naphthaquinone). These results are consistent with previous reports regarding the anti-inflammatory activity of juglone [128].

## 6. Concluding Remarks

The data summarized in this article suggest that many compounds derived from natural products exert potent anti-inflammatory properties. Although the drugability of pure anti-inflammatory compounds extracted from natural products seems a complicated task, extracts and pure compounds of natural products may still open new venues for therapeutic interventions. Pharmaceutical companies will probably not express high interest and invest hugely in compounds that will be difficult to patent. Nevertheless, if proven efficacious and safe, the use of natural products-derived compounds should be advocated by policy makers and health authorities. Regular consumption of such products may become a successful and safe strategy to treat chronic inflammatory conditions.

## Figures and Tables

**Table 1:** Summary of selected review articles (2005–2016) reporting on the anti-inflammatory effects of plant products *.

Main Theme of the Article	Major Method(s) of Testing	Major Active Materials/Compounds	Main Effects on Inflammation *	Ref.
Brazilian plants	Inflammation induction in rats and mice (e.g., by carrageenan)	Plants parts, various extracts, chromatographic fractions	Significant inhibition of COX and 5-LOX activity; reduction in edema volume	[47]
Plant-based foods	Plant-based food consumption in humans	Carotenoids, flavonoids, phenolic acids, monoterpenes, sulfides	Reduction in C-reactive protein (CRP) and IL-6 levels; inhibition of NFκB	[48]
Plant natural products	Various in vitro and in vivo models of inflammation (e.g., lipopolysaccharide [LPS]-induced) and cancer	Polyphenols, capsaicin, curcumin, ascorbic acid, indol-3-carbinol, geraniol, sulphoraphane, gingerol, lycopene, deoxyelephantophin	Significant reduction in cytokines levels; inhibition of COX-2, iNOS, NFκB and STAT (signal transducers and activators of transcription) activity	[49]
Plant barks	Various inflammation models in vivo (e.g., carrageenan-induced paw edema)	Various extracts, oleanolic acid, polyphenols, coumarin, β-amyrin, ursolic acid, β-sitosterol	Significant inhibition of COX and iNOS activity; attenuation of paw edema	[50]
Medicinal plants (General)	Various inflammation models in vivo (e.g., carrageenan-induced paw edema)	Various extracts; ambrosanolide, betulinic acid, ardisiaquinone G, polyphenols and others	Inhibition of COX, iNOS, 5-LOX and PLA2 activity; attenuation of paw edema	[51]
Herbal drugs (medicinal plants)	Various in vitro and in vivo (animals) models of inflammation; clinical trials in humans including safety and efficacy measures	Detailed compound families (e.g., alkaloids, glycosides, terpenoids, resins, essential oils, fatty acids, flavonoids, polysaccharides, phenolic compounds, steroids, cannabinoids, glycoproteins)	Significant reduction in cytokines, LTs, PGs and NO levels; inhibition of COX, iNOS, 5-LOX, PLA2 and NFκB activity; in humans: analgesic effects in different pain states, reduction of edema, attenuation of inflammatory measures	[52]
Virgin olive oil	Various in vitro and in vivo models of inflammation; clinical trials in humans	The phenolic compound oleocanthal	Reduction in cytokines, CRP, LTs, and PGs levels; inhibition of COX, iNOS, 5-LOX and NFκB activity	[53]
Medicinal plants	Various in vitro and in vivo models of inflammation	Whole plant or parts; alkaloids, glycosides, essential oils, fatty acids, flavonoids, nyctanthic acid, phyllanthin, and many others	Analgesic effects and reduction of inflammatory measures	[54]
Mangrove plants	Various in vitro and in vivo models of inflammation	Various extracts, pure compounds such as: agallochaol O, eugenol, mimosol D, calophyllolide	Reduction in cytokines, LTs, NO and PGs levels; inhibition of COX, iNOS, 5-LOX and NFκB activity	[55]
Herbal plants	Various in vitro and in vivo models of inflammation	Various extracts, plants parts, resins	Significant reduction in cytokines, LTs, NO and PGs levels; inhibition of COX, iNOS and 5-LOX activity	[56]
Marine natural products from soft corals	Various in vitro and in vivo models of inflammation (e.g., LPS-induced inflammation)	Sesquiterpenoids, diterpenoids, steroids, ceramide, cerebrosides, and many others (a comprehensive review with 339 structures)	Reduction in cytokines, NO and PGs levels; inhibition of COX and iNOS activity	[57]
Marine natural products of algal origin	Various in vitro and in vivo models of inflammation (e.g., LPS-induced inflammation)	Various extracts and pure compounds such as neorogioltriol, (12*Z*)-*cis*-maneonene-D	Reduction in IL-6, TNF-α, NO and PGs levels; inhibition of COX, iNOS, NFκB and STAT activity	[58]
Ethnobotanical plants	Carrageenan-induced paw edema	Various extracts	Significant reduction in edema volume; effects were similar to those of other anti-inflammatory drugs such as valdecoxib, sulindac, aspirin, diclofenac, ibuprofen, phenylbutazone and indomethacin	[59]
Plant-derived compounds	Various in vitro and in vivo models of inflammation; pre-clinical tests and clinical trials in humans	Curcumin, colchicine, resveratrol, capsaicin, epigallocatechin-3-gallate, quercetin	Reduction in cytokines, LTs and PGs levels; inhibition of COX-2, 5-LOX and NFκB activity; in humans: attenuation of inflammatory measures such as CRP, IL-1β, IL-6, and TNF-α	[60]
Active organosulfur compounds in garlic	Various in vitro and in vivo animals models (LPS-induced inflammation); studies in human volunteers and pre-clinical studies	Ajoene, diallyl sulfide, diallyl disulfide, allylmethyl sulfide, *S*-allyl cysteine, alliin, allicin	Anti-inflammatory: reduction in PGs, NO, IL-1β, IL6 and TNF-α levels; increase in IL-10 levels; inhibition of COX-2, iNOS and NFκB activity Pro-inflammatory: opposite effects of the mentioned above	[61]
Active organosulfur compounds and extracts of garlic	Various in vitro and in vivo animals models (e.g., LPS-induced inflammation); studies in humans	Aqueous, oil, chloroform and n-hexane extracts, as well as compounds in previous raw	Anti-inflammatory: reduction in IL-1β, IL-6 and TNF-α levels; increase in IL-10 levels; inhibition of NFκB activity Pro-inflammatory: increase in NO, IFN-γ and TNF-α levels	[62]
Indian medicinal plants	Various in vitro and in vivo models	Polyphenols, lignans, anthraquinones, flavonoids, alkaloids, terpenoids, saponins, polysaccharides	Reduction in TNF-α and other cytokines levels; inhibition of PLA2 activity; general–anti-inflammatory, analgesic and anti-allergic effects	[63]
Marine diterpenoids	Various in vitro and in vivo models (e.g., LPS-induced inflammation)	Eunicellane, briarane, cembrane and other diterpenoids	Significant reduction in IL-6, TNF-α, NO, PGs and LTs levels; significant inhibition of COX-2, iNOS, 5-LOX and NFκB activity, some of the effects were comparable to those of anti-inflammatory drugs such as indomethacin	[64]
Citrus flavonoids	Various in vitro and in vivo animal models (e.g., LPS-induced inflammation), healthy human volunteers	Hesperidin and hesperetin—two major flavonoids of citrus	Reduction in IL-1β, IL-6, TNF-α, NO and PGs levels; inhibition of COX-2, iNOS and NFκB activity; reduction in plasma CRP levels in humans	[65]
Plant polyphenols	Various in vitro and in vivo animal models (e.g., LPS-induced inflammation)	Plant polyphenols such as curcumin, apigenin, quercetin, *E*-cinnamaldehyde and E-resveratrol	Reduction in IL-1β, IL-6, TNF-α, NO and PGs levels; inhibition of COX-2, iNOS and NFκB activity	[66]
Marine natural products	Various in vitro and in vivo animal models (e.g., LPS or carrageenan-induced inflammation)	Detailed structures of 35 marine compounds such as steroids, fatty acids, diterpenes, sesquiterpenoids, alkaloids and polysaccharides	Significant reduction in IL-1β, IL-6, TNF-α, NO and PGs levels; significant inhibition of COX-2, iNOS and NFκB activity	[67]
Medicinal plants	Various in vitro and in vivo animal models (e.g., LPS-induced inflammation)	Isogarcinol, andrograpanin, hinokitiol, tectorigenin, α-iso-cubebene, schisantherin A, psoralidin, formosumone A, isofraxidin, maslinic acid, mangiferin	Reduction in IL-1β, IL-6, TNF-α, NO and PGs levels; inhibition of COX-2 and iNOS activity	[68]
*Acacia catechu* (Mimosaceae); in most reviewed studies extracts of *A. catechu* were combined with extracts of *Scutellaria baicalensis*	LPS-induced inflammation in vitro (cell lines and primary cells); arachidonic acid-induced inflammation and edema in mice ear; randomized, double-blind trial in patients with osteoarthritis	Catethin, epicatechin, flavonoids	In-vitro studies in cells—a mixture of *A. catechu* and *S. baicalensis* significantly reduced mRNA levels of COX, IL-1β, TNF-α and IL-6, and decreased the activity of NF-κB in LPS-stimulated cells; ear edema in mice—a mixture of *A. catechu* and *S. baicalensis* significantly attenuated COX and 5-LOX activity in the ear; osteoarthritis patients—a blend of *A. catechu* and *S. baicalensis* extracts (500 mg/day) led to a significant reduction in joint pain intensity (the effect was stronger than that of naproxen 440 mg/day) and, on the other hand, significantly increased plasma levels of IL-1β and TNF-α	[69]

* In this table, the word “significant” indicates that the *P* value for the difference between the tested groups is less than 0.05 or even smaller.

**Table 2:** Summary of selected research articles reporting on the anti-inflammatory effects of plant products.

Extracting Solvent(s)	Major Method(s) of Testing	Plant Species	Main Effects on Inflammation *	Ref.
80% EtOH in H_2_O	Carrageenan-induced paw edema in rats (for assessing inflammation)	75 species of medicinal plants that grow in Italy	Four species caused a significant reduction in paw edema similar to that seen under treatment with indomethacin. Other species exerted a less prominent edema-reducing effect	[70]
H_2_O	Carrageenan-induced paw edema in rats	Five species of Costa Rican medicinal plants: *Loasa speciciosa*, *Loasa triphylla*, *Urtica leptuphylla*, *Urera baccifera*, *Chaptalia nutans*	Four species caused a significant reduction in paw edema, similar to that seen under treatment with indomethacin	[71]
10% EtOH in H_2_O	Hot-plate method for assessing analgesia; carrageenan-induced paw edema	*Portulaca oleracea* L. subsp. *sati*v*a* (Haw.) Celak	A significant reduction in paw edema and an analgesic effect, similar to that of diclofenac	[72]
H_2_O	LPS-induced inflammation in a macrophage cell line (RAW 264.7 cells)	*Portulaca oleracea* L.	A significant reduction in IL-6, TNF-α, NO and PGE2 levels; decrease in iNOS expression; effects were more prominent than those of indomethacin	[73]
70% MeOH in H_2_O	Carrageenan-induced paw edema in chicks	*Portulaca oleracea* L.	A significant dose-dependent reduction in paw edema which was stronger than that seen under treatment with aspirin	[74]
*n*-Hexane, CHCl_3_, MeOH	Croton oil-induced ear edema in mice	*Sal*v*ia officinalis* L. (main active component is ursolic acid)	*n*-Hexane and CHCl_3_ extracts prominently reduced ear edema; MeOH extract had a weak effect while the essential oil was ineffective; the significant effect of ursolic acid was 2-fold stronger in reducing the edema than indomethacin	[75]
H_2_O, *n*-BuOH	Hot-plate method in mice; cotton pellet granuloma and carrageenan-induced paw edema in rats	*Sal*v*ia officinalis* L.	The H_2_O and BuOH extracts had a marked analgesic effect; both extracts significantly and dose-dependently reduced pellet granuloma and paw edema-effects were comparable to those of indomethacin	[76]
CHCl_3_, MeOH, EtOAc, *n*-BuOH	Carrageenan-induced paw edema in mice	*Salvia fruticosa*	A significant reduction in paw edema similar to that seen under treatment with diclofenac	[77]
H_2_O	Carrageenan-induced paw edema and yeast-induced pyrexia in rats	*Corchorus olitorius*	A significant reduction in paw edema which was stronger than that of aspirin; attenuation of hyperthermia (fever)	[43]
EtOH	Carrageenan-induced paw edema and cotton pellet-induced granuloma in rats	*Carica papaya*	A significant reduction in paw edema and pellet granuloma; effects were similar to those of indomethacin	[78]
MeOH	In-vitro assays for measuring neutrophils inflammation and lipoxygenase activity	*Vitex agnus-castus;* 10 compounds were extracted	Three compounds had a significant anti-inflammatory activity; two compounds inhibited the activity of lipoxygenase	[79]
Essential oils	LPS-induced inflammation in RAW 264.7 cells	*Origanum ehrenbergii* Boiss, *Origanum syriacum* L.	*O. ehrenbergii* caused a significant decrease in NO production	[80]
H_2_O	Ethyl phenylpropiolate and arachidonic acid-induced ear edema, carrageenan-induced paw edema, and cotton pellet-induced granuloma in rats	*Phyllanthus emblica* Linn.	Significant inhibition of ear inflammation and a reduction in paw edema and pellet granuloma—effects were similar to those of aspirin; the extract exerted an analgesic effect	[81]
MeOH	LPS-induced inflammation in RAW 264.7 cells	*Citrus paradis, C. limon* (L.) *Bur, C. kotokan Hayata, C. sinensis* (L.) *Osbec, C. reticulata Blanco, C. reticulata x C. sinensis, C. tankan Hayata*	A significant, dose-dependent reduction in PGE2 and NO levels; a significant decrease in COX-2 and iNOS expression	[82]
MeOH	Acetic acid-induced writhing in mice; carrageenan-induced paw edema in rats	*Mangifera indica*	A non-significant reduction in paw edema; a significant analgesic effect similar to that of diclofenac	[44]
MeOH	Hot-plate method in mice; cotton pellet granuloma and carrageenan-induced paw edema in rats	*Urginea indica* Kunth	Anti-inflammatory and analgesic effects, a significant reduction in paw edema; effects were similar to those of ibuprofen	[83]
70% MeOH in H_2_O, then, in different solvents	LPS-induced inflammation in RAW 264.7 cells	*Crataegus pinnatifida* Bunge var. *typica* Schneider	The aqueous extract caused a significant reduction in NO levels; and, a significant dose-dependent decrease in COX-2, IL-1β, IL-6 and TNF-α expression	[45]
H_2_O, EtOH	Inflammation induced by LPS and INF-γ in RAW 264.7 cells	40 Chinese plant species	Several extracts caused a significant reduction in NO and TNF-α levels	[84]
H_2_O, EtOH	Inflammation induced by LPS and INF-γ in J774A.1 cells	13 Chinese plant species and two fungi	Some extracts caused a significant reduction in NO and TNF-α levels	[85]
EtOH	Carrageenan-induced paw edema in rats	*Desmodium gangeticum*	A significant reduction in paw edema	[86]
*n*-Hexane, EtOAc, CHCl_3_, EtOH	Cotton pellet granuloma and carrageenan-induced paw edema in rats	*Vitex negundo* Linn; only the ethanolic extract was tested for biological activity	A significant decrease in paw edema and a modest reduction in pellet granuloma; effects were similar to those of indomethacin	[87]
EtOH	Carrageenan-induced paw edema in rats	*Sonerila tinnevelliensis* Fischer	A significant decrease in paw edema which was similar to that of indomethacin	[88]
MeOH	Hot-plate test & acetic acid-induced writhing in mice; carrageenan-induced paw edema in rats; yeast-induced pyrexia in rats	*Mentha spicata* L.	Significant dose-dependent analgesic effect, anti-inflammatory effect (reduction in paw edema) and antipyretic effect; effects were similar to those of reference drugs such as ketorolac and paracetamol	[89]
H_2_O	Carrageenan-induced paw edema in mice	*Micromeria fruticosa*	A significant reduction in paw edema; effect was similar to that of indomethacin	[46]
EtOH	12-O-tetradecanoylphorbol-acetate-induced ear edema in mice	*Malva sylvestris* L	A significant dose-dependent reduction in ear edema; a decrease in IL-1β levels and leukocytes migration to the tissue; effects were less potent than those of dexamethasone	[90]
Extra virgin olive oil	Acetic acid-induced writhing and formalin tests in mice; carrageenan-induced paw edema in rats	*Olea europaea*	A significant analgesic effect similar to that of aspirin; a significant reduction in paw edema similar to that seen under treatment with dexamethasone	[91]
80% MOH in H_2_O	Collagen-induced arthritis in mice	Polyphenol extract of extra virgin olive oil (*Olea europaea*)	A significant reduction in joint edema and bone loss; a significant decline in leukocytes migration; a decrease in PGE2, IL-1β, IL-6 and TNF-α levels; a significant reduction in COX-2 expression and NFκB activity, among other anti-inflammatory effects	[92]
EtOH and fractionation with *n*-hexane, CHCl_3_, EtOAc	*Aggregatibacter actinomycetemcomitans*-induced infection and inflammation in human oral cells (in vitro model)	*Malva sylvestris*	A significant reduction in protein levels of multiple pro-inflammatory mediators (e.g., IL-1β, IL-6, IL-8) and a decrease in their gene expression	[93]
EtOH	Assessment of 5-LOX activity in lung cancer cell line A549	*Abutilon indicum* L.	A significant reduction in 5-LOX activity	[94]
EtOH	Adjuvant-induced arthritis in mice	*Citrus x limon, Capsicum annuum* L.	A significant decrease in CRP, IL-1β, IL-6 and TNF-α levels; a significant reduction in arthritis	[95]
Acetone	LPS-induced inflammation in RAW 264.7 cells; assessment of 15-LOX activity	25 South African plant species	A significant reduction in NO levels; significant inhibition of 15-LOX activity	[96]
H_2_O	Carrageenan-induced paw edema in mice	*Morinda citrifolia* L.	A significant reduction in TNF-α levels; a significant decline in leukocytes migration; effects were comparable to those of indomethacin	[97]
EtOH and fractionation with *n*-hexane, CH_2_Cl_2_, EtOAc	Carrageenan-induced paw edema in mice	*Solanum lycocarpum* A. St. Hil.	A significant reduction in paw edema which was similar to that seen under treatment with indomethacin	[98]
EtOH then petroleum ether	Human peripheral blood cells stimulated with different antigens	*Azadirachta indica, Acacia catechu, Salmalia malabarica* (terpenoids were extracted)	A significant dose-dependent reduction in NO levels; a decrease in leukocytes count	[99]
MeOH	LPS-induced inflammation in RAW 264.7 cells; dextran sulfate sodium-induced colitis in mice	*Rosmarinus officinalis* L.	A significant dose-dependent decrease in nitrites, IL-6 and TNF-α levels; a significant reduction in COX-2 and iNOS expression; a significant decline in NFκB activity, among other inflammatory markers that were attenuated	[100]
90% EtOH in H_2_O	LPS-induced inflammation in human gingival fibroblasts	*Eriodictyon angustifolium*, 8 active compounds were extracted	A significant reduction in IL-6, IL-8 and MCP-1 levels	[101]
70% MeOH in H_2_O	LPS-induced inflammation in RAW 264.7 cells	*Drosera burmannii* Vahl. (insectivorous herb, sundew)	A significant dose-dependent decrease in nitrites and TNF-α levels; a significant dose-dependent reduction in COX-2 and iNOS expression	[102]
*n*-Hexane, CH_2_Cl_2_, EtOAc, MeOH	Carrageenan and experimental trauma-induced paw edema in mice and rats	*Ceratonia siliqua* L.	A significant dose-dependent reduction in paw edema which was similar to that seen under treatment with indomethacin	[103]
EtOH, acetone	LPS-induced release of TNF-α in THP-1 cells	Fourteen non-toxic extracts derived from six plants: *Cuphea carthagenensis* (Lythraceae), *Echinodorus grandiflorus* (Alismataceae), *Mansoa hirsuta* (Bignoniaceae), *Ouratea semiserrata* (Ochnaceae), *Ouratea spectabilis* and *Remijia ferruginea* (Rubiaceae); three non-toxic active compounds were extracted from *O. semiserrata*: epicatechin, lanceoloside A and rutin	Seven active extracts significantly reduced (>80% inhibition) TNF-α production. The effects of the extracts were comparable to that of dexamethasone (0.1 μM); epicatechin, lanceoloside A and rutin significantly decreased the release of TNF-α by approximately 67%, 65% and 42%, respectively	[104]
CH_2_Cl_2_, EtOAc, MeOH	Ear edema in mice; carrageenan-induced paw edema in rats; acetic acid-induced abdominal writhing and alteration of vascular permeability in mice	*Urera aurantiaca Wedd.* (Urticaceae)	A significant reduction in ear edema and myeloperoxidase activity in mice and rats (effects were less potent than those of indomethacin); a significant decrease in vascular permeability in mice (effect was comparable to that of indomethacin); a significant anti-nociceptive effect in mice which was comparable to that of indomethacin	[105]
MeOH	LPS-induced inflammation in RAW 264 cells	*Angelica acutiloba*	A significant decrease in NO, PGE2, IL-6 and TNF-α levels; a significant increase in heme oxygenase-1 expression, suggesting enhanced anti-inflammatory activity	[106]
H_2_O, EtOH	A testosterone-induced benign prostatic hyperplasia model in obese rats	*Serenoa repens*	A significant reduction in IL-1β, IL-6, NO and TNF-α levels	[107]
EtOH in H_2_O	Formaldehyde and adjuvant-induced Arthritis in rats	*Picrorhiza kurroa*	A significant reduction in synovial expression of IL-1β, IL-6 and TNF-α; a significant decrease in paw edema; a significant decline in NO levels and leukocytes infiltration to the inflamed joints; all the effects were comparable to those of indomethacin	[108]

* In this table, the word “significant” indicates that the *P* value for the difference between the tested groups is less than 0.05 or even smaller.

**Table 3:** Summary of selected reports of anti-inflammatory activity of pure compounds.

Compound(s)	Major Method(s) of Testing	Plants with High Concentration of the Compound(s)	Main Effects on Inflammation	Ref.
Triterpenes: α/β-amyrin acetate, nimbin, filicene, oleanolic acid	Carrageenan and formaldehyde-induced paw edema in rats	*Thymus serpyllum, Syzygium aromaticum, Salvia triloba, Rosmarinus officinalis, Origanum majorana, Ligustrum lucidum, Lavandula latifolia*	A significant reduction in edema volume; effects were comparable to those of hydrocortisone	[112]
Quercetin	Adjuvant and carrageenan-induced arthritis in rats (acute and chronic designs)	*Allium cepa, Camellia sinensis, Hypericum perforatum, Podophyllum peltatum*	A significant reduction in edema volume both in the acute and chronic models; effects were comparable to those of phenylbutazone	[113]
Allicin	Carrageenan-induced paw edema in rats	*Allium sativum* (garlic)	A significant reduction in edema volume which was similar to that of diclofenac	[114]
(−)-Myrtenol	Various models in mice: paw edema induced by various compounds, and, carrageenan-induced peritonitis (inflammation); acetic acid-induced writhing, hot-plate test, and, paw licking induced by formalin, glutamate, and capsaicin (nociception)	*Tanacetum vulgare, Aralia cachemirica*	A significant reduction in edema volume comparable to that of indomethacin; a significant decrease in IL-1β levels; a significant decline in leukocytes count; an significant analgesic effect which was comparable to that of morphine in most tests	[115]
Various terpenes and polyphenols	Inflammation induced by LPS in bone marrow derived dendritic cells	*Nepenthes mirabilis* (Lour.) Rafarin (Carnivorous plant)	A significant reduction in IL-6, IL-12 and TNF-α levels	[116]
Ferulic acid	LPS-induced inflammation in macrophages (in-vitro)	*Solanum lycopersicum* L. (Tomato)	A significant decrease in IL-1β and TNF-α expression; a significant reduction in NFκB activity	[117]
3-Hydroxyanthranilic acid	LPS-induced inflammation in RAW 264.7 cells and in mouse peritoneal macrophages	*Hibiscus tilliaceus*	A significant decrease in NO, IL-1β, IL-6 and TNF-α expression; a significant increase in IL-10 expression; a significant reduction in NFκB activity	[118]
